# Willingness and preparedness to provide care: interviews with individuals of different ages and with different caregiving experiences

**DOI:** 10.1186/s12877-021-02149-2

**Published:** 2021-03-25

**Authors:** Lea de Jong, Jona Theodor Stahmeyer, Sveja Eberhard, Jan Zeidler, Kathrin Damm

**Affiliations:** 1 Center for Health Economics Research Hannover (CHERH), Leibniz University Hannover, Otto-Brenner-Str.7, 30159 Hannover, Germany; 2 Health Services Research Unit, AOK Niedersachsen, Hannover, Germany

**Keywords:** Home care, Qualitative, Long-term care, Information, Older adult care, Content analysis, Willingness to care

## Abstract

**Background:**

At present, the provision of informal care to older relatives is an essential pillar of the long-term care system in Germany. However, the impact of demographic and social changes on informal caregiving remains unclear.

**Methods:**

Thirty-three semi-structured interviews were conducted with care consultants, informal caregivers and people without any caregiving experience to explore if people are willing to provide older adult care and how prepared these are with regard to the possibility of becoming care dependent themselves.

**Results:**

In total, three main categories (willingness to provide care, willingness to receive care and information as preparation) with several sub-categories were identified during the content analysis. While almost all interviewees were willing to provide care for close family members, most were hesitant to receive informal care. Other factors such as the available housing space, flexible working hours and the proximity of relatives were essential indicators of a person’s preparedness to provide informal care. It is, however, unclear if care preferences change over time and generations. Six out of 12 informal caregivers and nine out of 14 care consultants also reported an information gap. Because they do not possess adequate information, informal caregivers do not seek help until it is too late and they experience high physical and mental strain. Despite the increased efforts of care consultants in recent years, trying to inform caregivers earlier was seen as almost impossible.

**Conclusions:**

The very negative perception of caregiving as a burden was a reoccurring theme throughout all interviews and influenced people’s willingness to receive care as well as seeking timely information. Despite recent political efforts to strengthen home-based care in Germany, it remains unclear whether political efforts will be effective in changing individuals’ perceptions of informal caregiving and their willingness to be better prepared for the highly likely scenario of having to care for a close relative or becoming care dependent at a later stage in life.

**Supplementary Information:**

The online version contains supplementary material available at 10.1186/s12877-021-02149-2.

## Background

Because of recent demographic and social developments, older adult care has emerged as an important and widely debated topic worldwide. Similarly, in Germany media coverage of and public awareness about older adult and long-term care as well as related issues has increased over the last few years. Particular focus is given to current demographic forecasts, a shortage of qualified personnel, and concerns about the quality and financing of care services and personnel [1]. In recent times, political efforts have been taken to address these concerns by introducing care reforms and pursuing other objectives to strengthen this sector. For instance, the new definition of care dependency, which was introduced in 2017, resulted in an increase in the number of individuals who were classified as long-term care beneficiaries, and long-term care centers have been established to provide local support and disseminate information [2].

As part of the social security system in Germany, long-term care insurance is mandatory since 1995 and covers the entire population. Long-term care insurance funds are either linked to a statutory or private health insurance and entitlement to insurance benefits is based on a calculated care dependency level [3]. To assess the long-term care insurance entitlements of individuals, five care grades were introduced in 2017 to replace the previously used three care levels. The new instrument uses six modules (e.g. mobility, cognitive and communicative capacity or self-sufficiency) to determine the need for care of each person. Each of the six modules is comprised of a series of criteria, for which points are allocated. To classify a care grade, the points of all modules are added to a sum score, adjusted for the respective weight of the module. A range from 0 to 100 points is used for this classification. A higher care grade translates to a more severe care dependency [4]. Of the 2.8 million long-term care insurance claimants aged 65 years and above in 2017, 73% received home care [5]. Home care is either supported by cash benefits, in-kind benefits or a combination of the two. Cash benefits are usually passed on to informal caregivers with no regulations on how to use such benefits (given that adequate caregiving is guaranteed). In-kind benefits may be used for different types of professional home-care services. Expenses are covered by the long-term care insurance until the benefit cap of the respective care grade [6].

In a majority of home-based care settings in Germany, one or more informal caregivers are actively involved in providing or organizing care for the person who requires it. When family members, relatives, and/or friends assume the role of a caregiver, they often witness major changes in their everyday lives. A study by Geyer (2016) has shown that caring for a person in need for more than 1 h a day reduces working hours by approximately 5 to 8 h per week [7]. Further, such individuals may not be able to reenter the workforce as full-time employees once the caregiving situation has ended [8]. In addition, several studies have highlighted the heavy burden that caregivers shoulder. More specifically, informal caregiving adversely affects their physical and mental health and can lead to financial hardship [9, 10]. However, caregiving can also confer positive effects such as increased self-esteem, resilience, and meaning in relation to care provision [11, 12].

It is often reported that most older adults prefer to stay in their own homes or familiar surroundings for as long as possible. Nevertheless, research has shown that changing family dynamics and structures, such as increasing employment rates of women, higher number of single households and growing geographical distances between family members, can make informal and home-based caregiving more difficult [13, 14]. The exact reasons for people to take on the often time-consuming role of caregiver are complex and still not well understood [15]. When trying to understand the provision of informal caregiving, studies have focused on different determinants and used varying concepts. In a study by Broese van Groenou and De Boer (2016) the informal care model was applied as a theoretical foundation, in which dispositional factors and external conditions are of particular importance. The individual disposition of caregivers is further described by the person’s attitudes and affection, which incorporate individual’s motivation, values and normative beliefs concerning informal caregiving. Competence, time or financial resources, can be perceived as barriers to the individual disposition of caregivers [16]. Research on motivation behind informal care provision is largely theory-driven, with individuals explaining their reasons to take on the role of informal caregiver by for instance altruistic behavior or strategic exchanges in the form of money transfers [17]. Other studies have found that some caregivers feel a strong sense of responsibility and obligation to take care of older family members, others motivate their decision with feelings of love and reciprocity [15, 18]. The concept “motivation” was also investigated by a recent literature review from the perspective of self-determination theory. According to the study, motivation is important for the way in which caregivers ultimately experience informal caregiving [19].

Another group of studies focused on people’s willingness to provide informal care. In a report by the European Commission, the willingness of caregivers to provide care was singled out as a key determinant in terms of the availability of informal care. In addition, the extent of a person’s willingness to provide care with regard to, amongst others, the amount of hours per week is essential to determine the availability of informal care [20]. When it comes to people’s willingness to provide and receive or accept informal care, studies in Germany and the Netherlands have found the proximity of children, having a partner (or not) and having few siblings to constitute decisive indictors [21, 22].

Older adults are likely to become care dependent or have a relative who requires care as they grow older. In most cases, the question is not if, but how and when, such a situation will arise. Nevertheless, when a family member or close friend/acquaintance becomes care dependent, the need for support, consultation, and information gains significance [23, 24]. In addition, research has shown that family members who felt ready and prepared to take on different caregiving tasks, were generally prone to lower levels of mental and physical strain. Feelings of preparedness or readiness to care may therefore protect informal caregivers from high levels of burden and stress [25–27].

Accordingly, the aim of this qualitative study was to explore if people are *willing* to provide informal care and how *prepared* they are to care for a care-dependent person in the German context. Depending on the age of the interviewee, this can either primarily refer to an own hypothetical care dependency or having to take care of a care-dependent relative. The overarching research questions were 1) “Are people willing to provide informal care and which motives and other influencing factors play a role in the consideration?”, 2) “When providing or being willing to provide care, what are the expectations or wishes for receiving care?”, 3) “How prepared are individuals to provide informal care and what value does “preparedness to care” have for people?”

## Method

### Design

A qualitative explorative study was performed to gain insight into individuals’ perceptions of caregiving as well as reasons linked to their willingness and preparedness to provide informal care to older family members. Semi-structured face-to-face interviews were conducted with three different groups of individuals: (1) those with no prior caregiving experience, (2) informal caregivers, and (3) care consultants. Because of differences in their caregiving experiences, the perspectives of all the participants (i.e., all three groups) offered valuable insights into their willingness and preparedness to provide informal care. Parts of the results of this study have been reported elsewhere [28].

### Participants

Purposive sampling was used to recruit participants from self-help groups, long-term care centers in the region, and long-term care insurances [29]. Those with no prior caregiving experience were approached via direct email recruitment (personal contacts) with the appeal to forward the email to potential participants. Additionally, referrals for further participants were made by those already in the sample, in line with snowball sampling [30]. Prior to each interview, the participant was provided with sufficient information about the aims of the study, structure of the interview, data management procedure, and that their participation was voluntary.

Those who were ≥ 18 years of age, fluent in German, and able to provide informed consent were eligible for inclusion. The only other eligibility criterion that had been stipulated in advance was caregiving (in)experience. Specifically, to be eligible for inclusion, informal caregivers were required to be providers of care and/or assistance (either currently or in the past) to a person who required care in a home-based setting. In contrast, care consultants needed to have first-hand experience in providing consultation services to informal caregivers and people who are in need of care. Some care consultants also had personal experience in providing care to a person in need. Those individuals without any caregiving experience were deemed eligible for inclusion if they had no personal experience of providing informal care or assistance to a person in need of care.

Those with no prior caregiving experience (*n* = 7), informal caregivers (*n* = 12), and care consultants (*n* = 14) were interviewed between April and September 2018. On average, the interviews lasted for approximately 35 min, but the interviews with informal caregivers took considerably longer (between 30 and 75 min). The sample consisted of 11 men and 22 women. A majority of those with no prior caregiving experience were between the ages of 20 and 39 years. The majority of the care consultants and informal caregivers were between the ages of 40 and 59 years and 50 and 69 years, respectively. Care consultants are usually the first points of contact whom informal caregivers approach to acquire pertinent information (e.g. assessment procedure to obtain a care dependency grade, financial benefits, type and access to services). Therefore, from their vantage point, they can offer a comprehensive overview of caregiver needs and challenges. Since 2009, every person in need of care is entitled to a free care consultancy session provided by their long-term care insurance. Next to the statutory and private long-term care insurances, approximately 450 long-term care centers, more than 4.500 charity organizations and the majority of the 14.000 ambulatory services offer care consultancy services in Germany [5, 31–33]. Because of the heterogeneity of care consultants in Germany, different types of consultants were interviewed for this study. Three representatives from long-term care centers and consultancy centers each had been providing local support to informal caregivers and those who require care within the federal state of Lower Saxony. Five representatives from long-term care insurance companies and two commercial care consultants provided relevant inputs regarding the application, scope, and possibility of utilizing the existing care services within the German care infrastructure. Additionally, a lobbyist for informal caregivers was interviewed to gain insights into caregivers’ needs, wishes, and challenges. The lobbyist is part of an advocacy group that tries to strengthen the voice of informal caregivers in different settings (research, politics, other social communities). All but three care consultants had personal experiences in caregiving. All of the informal caregivers were either currently caring for a relative or had cared for a relative in the past, and a few of them had also provided care to a relative of their partner or close friends. All five care grades according to the long-term care insurance entitlements in Germany [4] were represented, and the duration for which they provided care ranged from 5 months to 20 years. For further details, please see Table 1.

**Table 1:** Description of included respondents (*n* = 33)

Perspective	No caregiving experience	Informal caregiver	Care consultant				
			Long-term care support centre	Consultancy centre	Long-term care insurance	Commercial care consultant	Lobbyist for informal caregivers
**n**	7	12	3	3	5	2	1
**Sex, n**							
Female	5	9	2	3	2	1	
Male	2	3	1		3	1	1
**Age (years), n**							
20–29	2						
30–39	4					1	
40–49			1		1		
50–59	1	2	1	3	4	1	
60–69		6	1				
≥ 75		4					1
**Family status, n**							
Single	2	1					
Married or in serious relationship	5	10					
Widowed		1					
**Children, n**							
Yes	5	11					
No	2	1					
**Employment status, n**							
Student	1						
Working, full-time	3	1					
Working, part-time	3	1					
Unemployed		2					
Retired		8					
**Relationship to the person in need of care, n**							
Relatives of mine		8	2	2	3		
Relatives of my partner						1	
Relatives of mine and relatives of my partner		3					
Relatives of mine and close friends		1			1		1
Relatives of mine, Relatives of my partner and close friends				1			
**Care grades, n**							
1		2					
2		2	1			1	
3		5	1	2	3		
4		3					1
5				1	1		
**Total care duration, n**							
Less than 1 year			1		1		
Between 1 and 2 years		3		1	1	1	
Between 3 and 4 years		1	1	1			
Between 4 and 5 years		2			1		
Between 5 and 6 years		2					
Between 8 and 9 years		2					
More than 10 years		2		1	1		1

Care consultants were only asked to provide information on their age, sex and the type of care consultancy they work at. Informal caregivers and people without any prior caregiving situation were additionally asked to provide information on their family status, whether they have children and employment status. Thus, for these three characteristics, information is only available for *n* = 19 interviewees. For the last three characteristics describing the respective caregiving experience of respondents, information is only available for *n* = 23 interviewees

### Procedure

Thirty-three semi-structured face-to-face interviews were conducted in the region of Lower Saxony, Northern Germany. To ensure homogeneity, one researcher (de Jong, M.Sc.) conducted all the interviews in German, and a native speaker translated the relevant excerpts into English for inclusion in this article (Translation Agency Perfect). All interviews were conducted in a neutral environment or, if preferred, in the home of the respondent. Based on literature reviews and expert discussions, an interview guide was developed for each group of participants (no caregiving experience, care consultants, informal caregivers). After each interview, the questions were revised based on newly emergent themes to incorporate the newly acquired knowledge. On average, six questions were posed to each interviewee, and several sub-questions were posed when required.

### Interview guide

With regard to those with no prior caregiving experience, the questions aimed to capture their notions about informal caregiving, willingness to provide informal care, and personal preferences regarding receiving care themselves, if they were to become care dependent. Informal caregivers were asked to describe their current or past caregiving experiences and delineate the factors that influenced their decision to take on the role of a caregiver, the challenges that they faced, and its effects on various aspects of their lives (e.g., occupation, finances, time for hobbies/family/friends). Informal caregivers were also asked to state (a) their personal preferences regarding care reception, if they were to become care dependent, and (b) any perceived differences in their preferences regarding care provision and reception. Care consultants were asked to describe the concerns that were most frequently shared by their clients and the challenges that informal caregivers commonly experience. The participants of all three groups were asked to indicate whether they considered care provision to be a familial or societal obligation.

### Data analysis

**Fig. 1:**
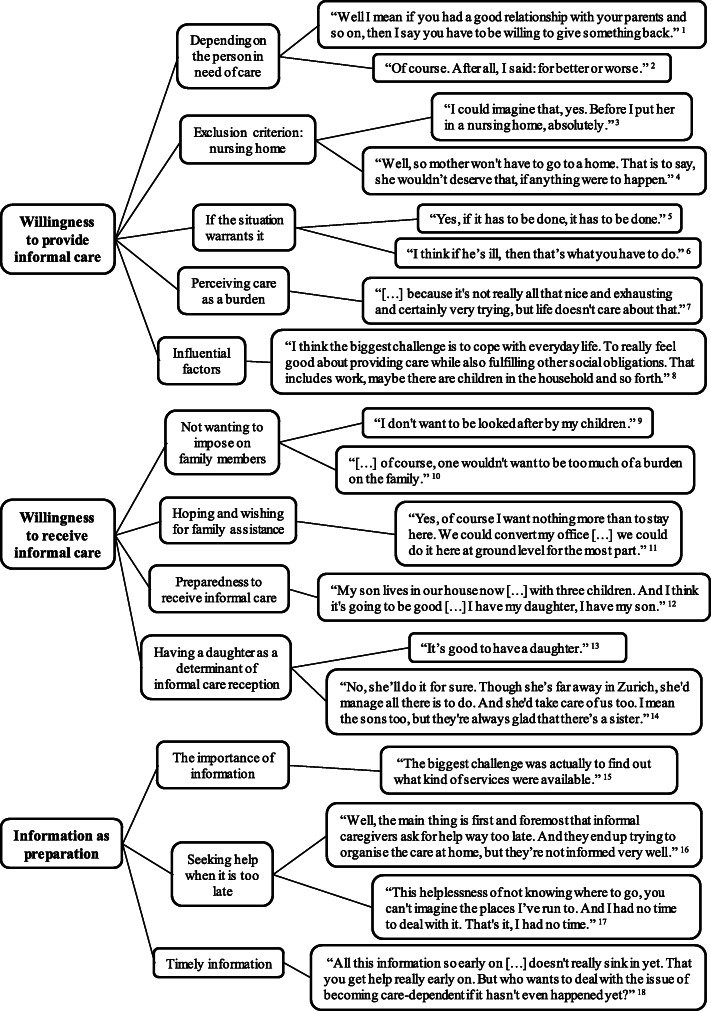
An overview of identified categories, subcategories and examples of characteristic participant responses. Legend: 1 = Informal caregiver 5. 2 = Informal caregiver 6. 3 = No caregiving experience 6. 4 = Informal caregiver 5. 5 = No caregiving experience 3. 6 = Informal caregiver 6. 7 = No caregiving experience 3. 8 = Care consultant 6. 9 = Informal caregiver 6. 10 = No caregiving experience 6. 11 = Informal caregiver 7. 12 = Informal caregiver 1. 13 = Informal caregiver 8. 14 = Informal caregiver 8. 15 = Informal caregiver 3. 16 = Care consultant 3. 17 = Informal caregiver 1. 18 = Care consultant 3

With the informed consent of each participant, all the interviews were audio-recorded and transcribed verbatim. To increase rigor, the transcripts were checked and verified for accuracy. Two researchers (de Jong and Damm) independently coded each transcript using MAXQDA version 11 and conducted a qualitative content analysis or, more specifically, a structured content analysis, in accordance with the procedure that has been outlined by Mayring [34]. Both researchers used deductive main categories, which were derived from the interview guide, and further inductive sub-categories were generated during the coding process from the interview material (for more details, see Fig. 1). Several overarching themes were identified and discussed during the coding process. Once the two researchers finished coding the transcripts, the two documents were merged and compared. Discrepancies were discussed among the team members, and required amendments were made.

### Ethical considerations and quality control

The Institutional Review Board of the Medical School in Hanover granted ethical approval to conduct this study (reference number: 09.05.17/La). A written informed consent form was signed by each participant prior to the interview. The quality of this study was checked with the COREQ (Consolidated criteria for reporting qualitative research) checklist, consisting of 32 items and aiming to improve the reporting of qualitative studies [35].

## Results

In total, three main categories and several subcategories were identified using the content analysis. An overview of the categories is provided in Fig. 1, and a detailed description of each category is presented in the following sections. It needs to be noted here that the statements in the category “willingness to receive informal care” were not made by care-dependent people themselves that are currently receiving care but rather are opinions of respondents concerning their hypothetical willingness to receive informal care.

### Willingness to provide informal care

Over the course of the interviews, each participant was asked to indicate his or her willingness or readiness to take on the role of a caregiver. Depending on their caregiving experience, they provided responses based on hypothetical scenarios or current or past caregiving experiences. Being willing to care was contingent on several factors such as the nature of the relationship that they shared with the care-dependent person or the need to exclude other undesirable options (e.g., admission to nursing homes).

#### Depending on the person in need of care

When asked about their willingness to provide care, the responses of all the young interviewees (20 to 39 years of age) with no prior caregiving experience were brief but positive. The responses of those who belonged to the other two groups (informal caregivers and care consultants) tended to be more complex, and their willingness was contingent on the nature of the relationship that they shared with the care-dependent person, the extent and type of support that he or she would require (e.g., caregiving time, tasks, and duration), and living arrangements. It remains unclear if the willingness to provide care changes as a function of age and/or caregiving experience. Since informal caregiving is a time-consuming activity, several participants in all three groups reported that their willingness to provide care would depend on the person that was in need of care, specifically, the nature of the relationship that they share with the care-dependent person. Most were willing to provide informal care to not only their partners but also their parents (mother or father).

“*Yes. For sure. Well, not for all of my relatives. But, for my own mother, I would definitely do that.*” (No caregiving experience 1)

They often attributed their willingness to care for their parents and closest relatives to love, reciprocity, and the desire to give something back to these individuals. However, this was observed only among those who shared close relationships with their parents and had positive childhood memories.

#### Exclusion criterion: nursing home

For several participants with no caregiving experience (20 to 39 years of age), their willingness to provide care was rooted in their unwillingness to admit their loved one to a nursing home and place the responsibility of caring for him or her in the hands of a stranger; thus out of one’s own control and quality standards. These arguments were often attributable to the negative manner in which nursing homes have been portrayed by the media and perceived by society.

“*Personally, I would find it difficult to just put her in a nursing home, into someone else’s hands.*” (No caregiving experience 2)

Several informal caregivers (50 to 69 years of age) reported similar reasons. For some, it was an acute care dependency (often hospital stay), in which the possibility of a nursing home was excluded right away and it was decided to assume the role of an informal caregiver. For others, equating the life in a nursing home with poor quality of life led them to take on the informal caregiving role. In addition to the reasons reported by those with no prior caregiving experience, informal caregivers noted that their negative perceptions stemmed from their memories of seeing their older friends or neighbors in their nursing homes.

#### If the situation warrants it

In contradistinction to the motives of love and reciprocity, several interviewees with and without caregiving experience attributed their willingness to provide care to a sense of obligation. Instead of offering a positive rationale (i.e., love for the person who requires care), they provided a negative reason. Caregiving was perceived to be a burdensome task, and such situations were described as life events that no one wishes for but have to be dealt with.

“*Of course. If it has to be done (laughs). But, I obviously wouldn’t be crazy about the idea*.” (No caregiving experience 3)

When delineating her reasoning behind wanting to provide informal care, one participant elaborated upon her sense of obligation. She considered her family to be her first priority. Therefore, if the situation warranted it, she was willing to accept the consequences of taking on this responsibility on her working hours and free time.

“*If it has to be done, then family comes first, and I would cut back in other areas of my life. I don’t know if, once it is foreseeable, we can find other options or ways or at least help from older adult care services and the like.*” (No caregiving experience 4)

Moreover, more than half of the interviewed informal caregivers also felt obliged to provide informal care, especially to their partners.

#### Perceiving care as a burden

Across all the interviews, the perception of caregiving as a burden was a frequently recurring theme, and it was closely linked to their willingness to provide care. Almost all of the informal caregivers perceived caregiving to be a burden because of the high levels of mental and physical strain that they had experienced. Even those with no prior caregiving experience perceived or, more accurately, imagined informal caregiving as something that no one wishes for, and they described it as a strenuous, burdensome, and trying task.

“*Not even necessarily, but I believe that* [providing informal care] *should not be underestimated. It is a burden, and you have to invest a lot, and I believe that this is also an environment where sorrow is quite evident.*” (No caregiving experience 5)

Their perceptions of caregiving were partly rooted in their own personal experiences (e.g., observing their parents care for their grandparent) or media portrayals.

#### Influential factors

The intrinsic desire to provide care may not necessarily translate into the actual provision of informal care. Accordingly, participants without caregiving experience (30 to 39 years of age) as well as informal caregivers underscored the different factors that are likely to influence their willingness to provide care. They did not consider their willingness to be an absolute state. Instead, they perceived it as one that is contingent on other personal responsibilities that can prevent them from being able to provide informal care. Examples of such responsibilities included the need to work to ensure financial stability, having young children at home, and large geographical distances between family members.

“*There are several prerequisites. Family must live in the same city. That is not the case for us at all. That wouldn’t work from here. I think there are professional and, as always, other factors that have an influence on that*.” (No caregiving experience 4)

Many informal caregivers also reported these personal responsibilities, especially when they explained why it was possible for them to provide informal care. For example, flexible working hours and the possibility of working from home were reported as factors that permitted them to provide informal care. Additionally, three informal caregivers reported that living in the same house or close to the relative who required care were the core foundational factors that made informal caregiving possible and a self-evident option.

“*Thank God we had our mother-in-law in the house! That is why the foundation—the foundation was already there*.” (Informal caregiver 1)

According to representatives of different types of care consultancies (long-term care insurance, commercial care consultants, long-term care support centers) and informal caregivers, large geographical distances between family members (e.g., those living in other countries or federal states) make it difficult for relatives to actively take on the role of a caregiver, when moving is either not possible or desired. Some informal caregivers who were willing to provide care had set clear boundaries with regard to the location in which they would provide care (i.e., own house or apartment), the type of care that they would provide (e.g., personal hygiene), and the number of hours (per week) for which they could provide care.

### Willingness to receive informal care

#### Not wanting to impose on family members

When asked about their preferences regarding care reception in a home-based setting, the participants, especially the younger ones who lacked prior caregiving experience (20 to 39 years of age), reported that they did not want to impose on their family members and concordantly perceived caregiving as a burdensome task. All of them were willing to provide informal care; however, they did not want to become a burden, especially to their own children or partner. The main reasons for such a preference were the toll that it takes on the caregiver and their unwillingness to restrict their lives to take on the role of a caregiver.

“*Because you do hear from other people caring for relatives at home that it’s a full-time job. Personally, I wouldn’t want to put my kids or my partner or whomever through that*.” (No caregiving experience 4)

Informed by their personal experiences, several informal caregivers reported that they did not want to impose on their own children. More specifically, one informal caregiver mentioned that having a full-time job and small children at home makes it almost impossible for a person to care for a loved one who requires care. Although she wished to stay in her own home for as long as possible, she did not want to impose on her own children. She reported that she would voluntarily move into a nursing home to spare them the burden of caring for her.

“*They all work. They won’t be able to do what I was able to do as a housewife. And, like I said, it’s hard as hell, and I’d go into a nursing home as well for the sake of the children. But, of course, it’s everyone’s wish to be properly cared for in their own homes, no question about that.”* (Informal caregiver 2)

In contradistinction to the relatively brief statements that were made by those with no prior caregiving experience, this response, which was provided by an informal caregiver, is an example of a more nuanced explanation. Informal caregivers mentioned that, in the future, it would be necessary to strike a balance between their wish to (a) live in a home-based setting rather than a nursing home for as long as possible and (b) not become a burden to their children and family members.

#### Hoping and wishing for family assistance

Most of the informal caregivers and care consultants wished or hoped to continue living in their own homes for as long as possible. The statements in this category were more cautious. They did not clearly describe their expectations about receiving informal care; instead, they articulated their wish to receive familial support in the future. For example, one care consultant offered a more nuanced explanation, wherein she differentiated between her expectations about receiving care from her children and her partner. Specifically, she stated that she would like her partner to play some role in ensuring that they continue living in their own home for as long as possible.

“*I have children too. I wouldn’t expect them to care for me. I don’t know what would be going on in their lives then; no idea. I have a partner. So, I would want to and expect to be at home with him for as long as possible.*” (Care consultant 1)

Another care consultant believed that anyone with a good relationship with his or her family members and relatives would want them to play some type of caregiving role when they find out that he or she requires care.

“[ … ] *but I think anybody who has any relatives or family would like to be supported and for them to take on some of it.*” (Care consultant 2)

None of the participants wanted any of their family members to be their sole caregiver. Instead, they wanted them to play a supportive role, but they differed in the extent to which they wanted them to play a supportive role.

#### Preparedness to receive informal care

A few informal caregivers articulated clearer expectations about receiving informal care from their family members in the future. Their houses were perceived as one of the key foundations that made independent home-based care possible. Accordingly, these informal caregivers had already made concrete plans to repurpose specific rooms and make the living space more accessible. Living in a ground-floor apartment and proximity to one’s child (e.g., living in the same house or close by) were considered to be indicators of preparedness for old age, because they facilitate the provision of home-based care, if and when necessary.

“*Of course. Thank God we have the possibility! My son lives right above us. We’re on the ground floor; so, we’ve already pretty much set everything up*.” (Informal caregiver 1)

In very few cases, expectations about receiving informal care had been clearly communicated through strategic exchanges. One participant reported that her parents had promised her sister their house in exchange for taking care of them when they need her assistance. This may also be partially attributable to differences in expectations between those who live in urban and rural areas.

“*For sure. I’m from the countryside, and I am one of four children—fortunately, at this point—and I live the farthest away from everyone. And* [my parents] *clearly expect that. They have a single-family house, which they have more or less gifted to my oldest sister, so to speak, with a promise that they will be taken care of. So yes, these are traditional expectations, as it were. They did it just like that with their parents*.” (Care consultant 1)

#### Having a daughter as a determinant of informal care reception

Although male interviewees were willing to provide care, several participants in all three groups considered daughters to be the person who is most likely to take on the role of a caregiver. Those who had a daughter often expressed a sense of relief because they knew that they would be taken care of, if such a need were to arise. Numerous informal caregivers had already spoken to their daughters about hypothetical situations in which they may be rendered care dependent and had shared their wishes with them. Therefore, they were able to rely on their support. In the case of several female informal caregivers the decision to take on a caregiving role was a self-evident option and not based on previous family discussions on responsibilities. In these situations, their brothers were also involved, but they tended to play a supportive role. However, in most cases, care-dependent mothers made this decision by first asking their daughters for help.

“*Yes, and my mother was getting older and needed more help. And there was really no question about who would do it. That was me.*” (Informal caregiver 3)

One female informal caregiver further distinguished between having sons (whom she had) and being her mother’s daughter and primary caregiver. For example, she described the composition of the support groups for relatives in the nursing home. Another informal caregiver, who was providing care to her partner, mentioned that her daughter had already assured them of her organizational support (if required), despite a large geographical distance between them. Further, she mentioned that, while her two sons would also support them, they were glad to have a sister on whom they could rely.

### Information as preparation

Care dependency creates a substantial need for information. Thus, in all the interviews, information emerged as a relevant theme. Information here refers to information about available support services, insurance benefits as well as what informal caregiving might entail or what should be expected. In particular, information was considered to be an indicator of adequate preparedness to provide care to a care-dependent person. The possibility of informing people about available support services at an early stage was additionally discussed.

#### The importance of information

As the likelihood of becoming care dependent increases with old age, it is no surprise to find yourself in such a situation. Nevertheless, more often than not, the participating informal caregivers (50 to 69 years of age) had gradually grown into their caregiving roles without much preparation or planning.

“*We never used to talk about that. It never crossed either of our minds, or let’s just say at least not my mind.*” (Informal caregiver 4)

In retrospect, however, all the informal caregivers criticized the lack of information about available care services and financial support. Further, the care consultants observed that, sometimes, the available information on insurance benefits and services does not reach those who need it the most. For example, one care consultant recounted a conversation between her and an informal caregiver in which the importance of information in facilitating and prolonging informal caregiving at home (with support) was emphasized.

“*We had a relative who said, ‘If only I had known this before.’ They took care of the mother for a couple of months, I think, and, then, she passed away. The daughter was so unhappy, because we could have prolonged this* [informal caregiving at home] *if she’d known sooner. That’s kind of the problem, isn’t it? There’s not enough information*.” (Care consultant 3)

The caregivers stated that, if they had known what informal caregiving would entail, they would have prepared better, also with respect to accessing home-based support services sooner instead of coping with the given circumstances for too long. On the one hand, information was considered to be essential in helping one prepare for a situation in which he or she might have to take on the role of a caregiver. On the other hand, informal caregivers highlighted the uncertainty surrounding the extent to which their support would be needed, and this made it difficult for them to adequately plan and prepare in advance.

#### Seeking help when it is too late

Based on the responses of the care consultants and informal caregivers, the unmet need for information on services and the extend of insurance benefits was identified as a major issue. Rather than challenges in accessing services, informal caregivers and consultants highlighted an information gap about available services. This is regarded as the main prerequisite for actually seeking help and accessing assessment and support services. Those who are confronted with the task of providing informal care are often inadequately informed about and prepared for this task. Even when a family member gradually becomes more dependent on external sources of support, individuals tend to cope with their current circumstances without additional help for as long as possible.

“*I think it’s safe to say that one generally addresses the matter too late. I believe that to be the problem behind it all. Even if you have parents or someone else, when you realize that things aren’t going too well, you look at them and say, ‘Yes, that’s still alright.’*” (Care consultant 4)

Therefore, informal caregivers often fail to recognize and cope with their high levels of mental and physical strain for a long time.

“*First of all, my health. I just couldn’t do it anymore. My nerves were at a breaking point and I was emotionally drained. I was really at the end of my strength*.” (Informal caregiver 6)

According to almost all care consultants, this is one of the reasons why many informal caregivers constitute the next generation of care-dependent individuals. More specifically, care consultants reported that informal caregivers find it difficult to let go of their own quality standards and strong sense of obligation to provide informal care and therefore fail to seek help early enough to avoid feeling overburdened.

#### Timely information

Although the likelihood of becoming care dependent or being required to care for a care-dependent relative increases with age, a majority of individuals prefer to not think about and prepare for such an event. Care consultants, therefore, try to provide timely information through different local information sessions or directly visit large firms and inform their employees. Half of the care consultants (long-term care support centers and insurance) underscored the need for early information, but they also noted that, if a person is not directly confronted with a situation in which he or she is required to provide care, he or she is unlikely to be receptive to the available information.

“*Well, I think it’s human nature to shun things and ignore things I don’t want to have to deal with. And, yet, we still try to pass out information material and set up campaigns in such a way, so as to give out information at a very early stage*.” (Care consultant 5)

At the same time, all of the long-term care insurance consultants observed that information culture is already quite widespread in Germany. Nevertheless, until a person is confronted with such a situation, he or she is unlikely to pay adequate attention to such information and prepare for such an event. Thus, providing early information is not the main challenge; instead, informing them at an appropriate time to ensure that they seek help by accessing available services before it is too late (high physical and mental strain) is a major challenge.

## Discussion

As it is often only a matter of how and when someone within one’s own family becomes care dependent, the aim of this qualitative study was to explore firstly if people are willing to provide informal care and secondly how prepared they are to care for a care-dependent person. At present, the provision of informal care to older relatives is an essential pillar of the long-term care system in Germany and many other countries [20, 36]. However, the impact of demographic and social changes (e.g., changing family structures and labor market participation) on informal caregiving remains unclear.

The first research question of this study investigated people’s willingness to provide care and its influencing factors. A vast majority of the interviewees were willing to provide informal care to close family members such as their partner and parents. Different motives were used by respondents to describe the reasons for their willingness to provide care. Whereas some participants attributed their willingness to provide care to motives of love and reciprocity, others attributed it to a sense of obligation and their desire to avoid admitting their loved ones to a nursing home. Age and experience of respondents tended to result in more complex statements with regard to their willingness to provide care. It remains, however, unclear if the willingness to provide care of our respondents changes as a function of age and/or caregiving experience as we did not follow our respondents over a period of time. Many participants stated that their future decisions to provide informal care were not certain and that their actual decisions would depend on other influential factors such as the available housing space, occupational demands, the geographical distance between family members, personal relationships, and having children at home. Some of the perceived barriers to caregiving found in our interviews were also reported in two recent studies, in which determinants of providing informal care were investigated. In particular, proximity of and having children and/or a partner at home [21] as well as having few siblings, a short geographical distance between relatives, and having a widowed parent without a new partner emerged as decisive factors [22].

In contrast, to answer our second research question, quite a few interviewees were less inclined to receive informal care. It needs to be reiterated that the opinions and wishes of the respondents were purely speculative, as none had personal experience of receiving informal care by family members. Such views can therefore change over time. In the interviews, the reasons of respondents for their reluctance in voicing their willingness to receive care ranged from not wanting to impose on family members to cautiously hoping to receive familial support and clearly expecting the support of their daughters. While individuals were generally willing to provide care, they did not expect the same in return. The desire to not impose on family members was most frequently reported by those who had no prior caregiving experience, and this preference was rooted in their negative perception of caregiving as a burden. It remains unclear whether this wish to not become a burden to one’s family members is a recent generational aspiration or if earlier generations also espoused the same wish but changed their views and care preferences when they grew older. With regard to expecting the support of one’s daughters, Sharma, Chakrabarti and Grover (2016) as well as Six, Musomi and Deschepper (2019) have reported similar results, still suggesting a traditional role for women to take care of their older relatives [37, 38]. It is noteworthy that it remains unclear whether one’s willingness to provide or receive care changes over time. Additionally, those who live in rural and urban areas may differ in their expectations about family members providing care to each other, and these differences need to be investigated further. Longitudinal studies should be conducted to examine the stability and consistency of long-term care preferences across time and generations.

Our third research question investigated people’s preparedness to care. Living in a ground-level apartment or in the proximity of children, especially daughters, were considered good indicators of preparedness for old age. At the same time, informal caregivers often reported not having prepared in advance, as it was difficult to plan for the intensity of care dependency and the future living arrangements of the care-dependent person and informal caregiver are often uncertain in advance. In addition, it is especially difficult for most individuals to think about and prepare for a situation in which they would be rendered care dependent. Nevertheless, recent statistics suggest that there has been an increase in the number of people who opt for supplementary insurance for long-term care. This trend may be attributable to the extensive supply of information in Germany, and it may be an indicator of improved foresight [39].

The possession of adequate information was considered to be a prerequisite for preparedness for caregiving and accessing available services. The (unmet) need for information was reported as a challenge by almost all of the informal caregivers and care consultants. Accordingly, in Germany, care consultants have tried to increase the dissemination of available information. However, informing people at an early stage (i.e., before a loved one becomes care dependent) was perceived to be almost impossible. Because informal caregiving is often perceived to be a burdensome task, most people try to not think about such a possibility, unless absolutely necessary. Additionally, preferences and available care options change over time, and the actual extent of care dependency remains uncertain. Thus, early information may become outdated by the time caregiving is warranted. However, because of the unmet need for information, many informal caregivers do not seek help until it is too late, and they experience a high level of mental and physical strain. Feelings of preparedness to care have been associated with decreased levels of burden and caregiver strain in previous studies [25]. Instead of trying to inform people earlier, it may be more prudent to ensure that new informal caregivers receive the requisite information at the right time. More specifically, it is very crucial that they know whom to contact to acquire the requisite information. According to the participating care consultants, the diversity of such care consultancies in Germany make it more difficult for new informal caregivers to correctly identify the service provider that they should approach to acquire the information that they need [28].

Beyond what was already said to the three research questions, we found that irrespective of the group to which they belonged, almost all the interviewees held negative perceptions of caregiving (i.e., when providing or receiving informal care) as a burdensome and demanding task. First, care consultants delineated the challenges that were faced by informal caregivers, and informal caregivers reported that they experienced mental overload and physical strain as a result of providing care. Second, most young participants with no prior caregiving experience perceived aging and informal caregiving very negatively. This perception was mainly rooted in negative media portrayals of older adult care (e.g., shortage of personnel and poor quality care provided in nursing homes) [1]. Although past findings suggest that caregiving does have positive effects, such as increased self-esteem [11], only one informal caregiver considered the task of caring for her care-dependent husband to be a positive experience. Positive perceptions of aging have been found to be positively associated with Singaporean teenagers’ willingness to care for their parents in the future [40]. In addition to perceptions of caregiving, the recognition of informal caregiving as an important and difficult task is vital and has increased over the past few years.

### Limitations

This study has several limitations, which need to be articulated. First, the sample was recruited from one federal state in Germany, namely, Lower Saxony. This limits the transferability of the findings. Although efforts were taken to maximize the heterogeneity of the participants, the findings cannot be generalized to the general population because the sample size was only 33. Moreover, despite our best efforts to recruit a higher number of male informal caregivers, a higher number of women participated in our study. Since our data were collected at a single point in time, temporal changes in preferences regarding care provision and preparedness to provide informal care could not be examined. Ideally, researchers should use longitudinal designs and large samples to examine the decision-making processes that underlie the process by which one’s willingness to provide care translates into the actual provision of informal care until a point is reached when alternative solutions have to be sought.

## Conclusion

In conclusion, this study provided insights into reasons for providing informal care, looking at people’s willingness to provide and receive care, diverging expectations and notions of preparedness to care. A person’s willingness to provide care was contingent on the (relationship to the) care-dependent person and explained by different motives. Willingness to provide care was not seen as an absolute state, but rather influenced by potential barriers such as distance between family members or time restrictions due to work or familial obligations. Willingness to provide care of our respondents was also highly influenced by the negative perception of caregiving as burdensome. While individuals were generally willing to provide care, the negative perception of caregiving and not wanting to impose on their own children often resulted in individuals being reluctant to receive care. The possession of adequate information emerged as an important indicator of one’s preparedness to provide care to care-dependent individuals. To avoid caregivers experiencing high physical and mental strain caused by caregiving, the need to provide timely information to potential caregivers was underscored. Nevertheless, despite the best efforts of the care consultants, providing early information (i.e., before the need to care for a care-dependent person arises) was perceived to be almost impossible. This was attributable (at least in part) to the very negative perceptions of caregiving that were held by our participants. Further research is needed to examine how long-term care preferences change across time and generations as well as on the best timing for providing information to (potential) informal caregivers in order to reach individuals and avoid significant strain.

## Supplementary Information

**Additional file 1.** Interview Guide.

## Data Availability

The data generated during the current study are not publicly available due to data protection regulations but are available from the corresponding author on reasonable request.
