# Supplementary material for: Willingness and preparedness to provide care: interviews with individuals of different ages and with different caregiving experiences
Source: BMC Geriatr. 2021 Mar 25;21:207. doi: 10.1186/s12877-021-02149-2 (PMC7992803; doi:10.1186/s12877-021-02149-2)
Supplement: Supplementary file 1 — Additional file 1. Interview Guide. [file 12877_2021_2149_MOESM1_ESM.docx]

| **Interview Guide** | |
| --- | --- |
| **Group: Individuals with no prior caregiving experience** | |
| **Main questions** | **Follow-up questions or comments** |
| 1. Have you ever thought about your wishes or preferences if you were to become care dependent when you grow older? 2. What do you think your relatives wish for in case of a care dependency situation? | - Have you ever talked to your relatives (e.g. your parents) about these wishes? |
| 1. Do you know what options are available for older adult care? | - Do you have a preference regarding these options if you were to become care dependent (alternative: for your relatives)? |
| 1. Could you imagine providing informal care to your relatives? 2. Would you be willing to provide informal care if this means that you have to 1) forgo part of your salary 2) reduce your working hours or 3) reduce your leisure time (time for hobbies, friends and family)? 3. Are there certain areas where you would prefer formal nursing assistance? | - Could you elaborate a bit more on your motivations behind this (lack of) willingness? |
| 1. Who do you think is responsible for providing or organizing care in the event of a care-dependency situation? | - Familial vs. societal responsibility |
| **Group: Informal caregivers** | |
| 1. How do you currently care for your relative (or retrospectively cared for your relative)? | - Do you make use of any professional services? |
| 1. Does your current care situation reflect your wishes? 2. Do you have the feeling your (care-dependent) relative would like something different? |  |
| 1. What factors played a role in your decision to take on the role of informal caregiver for your relative in need of care? | - What was the most important reason for you to take on this role/responsibility? |
| 1. What do you perceive to be major challenges in organizing and/or providing care to your relative? 2. Can you estimate how your everyday life, in particular your time distribution with regard to 1) working hours 2) time for leisure (family, friends, hobbies) 3) time for the person in need of care, has changed? 3. Does caring for your relative affect your financial situation? 4. In which areas would you like formal nursing support? |  |
| 1. What are your wishes if you were to become care-dependent? 2. Do your wishes differ in the case of an own care-dependency from those for your relative(s)? 3. Who do you think is responsible for providing or organizing care in the event of a care-dependency situation? | - In which areas would you prefer professional nursing assistance? - Have your wishes changed because of your caregiving experience? - Familial vs. societal responsibility |
| 1. If you think about the current long-term care system in Germany, do you have any concrete suggestions for improvement? |  |
| **Group: Care consultant** | |
| 1. What are the most common inquiries or concerns that are shared by informal caregivers? |  |
| 1. What are the most common challenges informal caregivers experience when organizing and/or providing care? | - Further sub-questions depending on the expertise of the care consultant. |
| 1. What are your wishes if you were to become care-dependent? 2. Do your wishes differ in the case of an own care-dependency from those for your relative(s)? 3. Who do you think is responsible for providing or organizing care in the event of a care-dependency situation? | - Do you have informal caregiving experiences? - In which areas would you personally prefer professional nursing assistance? - Have your wishes changed because of your caregiving experience and knowledge? - Familial vs. societal responsibility |
| 1. If you think about the current long-term care system in Germany, do you have any concrete suggestions for improvement? 2. Do you have any wishes regarding new reforms or changes to the law? |  |
